# Microbial Diversity in Decaying Oil Palm Empty Fruit Bunches (OPEFB) and Isolation of Lignin-degrading Bacteria from a Tropical Environment

**DOI:** 10.1264/jsme2.ME18117

**Published:** 2019-04-23

**Authors:** Analhuda Abdullah Tahir, Nor Farhana Mohd Barnoh, Nurtasbiyah Yusof, Nuurul Nadrah Mohd Said, Motoo Utsumi, Ang May Yen, Hazni Hashim, Megat Johari Megat Mohd Noor, Fazrena Nadia MD Akhir, Shaza Eva Mohamad, Norio Sugiura, Nor’azizi Othman, Zuriati Zakaria, Hirofumi Hara

**Affiliations:** 1 Department of Environmental Engineering and Green Technology, Malaysia-Japan International Institute of Technology, Universiti Teknologi Malaysia Jalan Sultan Yahya Petra, 54100 Kuala Lumpur Malaysia; 2 Graduate School of Life and Environmental Science, University of Tsukuba 1–1–1 Tennodai, Tsukuba, Ibaraki, 305–8572 Japan; 3 Analytical and Scientific Instruments Division, Shimadzu Malaysia Sdn. Bhd. Nouvelle Industrial Park 2, Taman Sains Selangor 1, Kota Damansara, 47810, Petaling Jaya, Selangor Malaysia; 4 Department of Mechanical Precision Engineering, Malaysia-Japan International Institute of Technology, Universiti Teknologi Malaysia Jalan Sultan Yahya Petra, 54100 Kuala Lumpur Malaysia; 5 Department of Chemical Process Engineering, Malaysia-Japan International Institute of Technology, Universiti Teknologi Malaysia Jalan Sultan Yahya Petra, 54100 Kuala Lumpur Malaysia

**Keywords:** microbial diversity, lignocellulose degradation, tropical region, lignin degradation

## Abstract

Oil palm empty fruit bunches (OPEFB) are the most abundant, inexpensive, and environmentally friendly lignocellulosic biomass in Malaysia. Investigations on the microbial diversity of decaying OPEFB may reveal microbes with complex enzymes that have the potential to enhance the conversion of lignocellulose into second-generation biofuels as well as the production of other value-added products. In the present study, fungal and bacterial diversities in decaying OPEFB were identified using Illumina MiSeq sequencing of the V3 region of the 16S rRNA gene and V4 region of the 18S rRNA gene. Fungal diversity in decaying OPEFB was dominated by the phylum *Ascomycota* (14.43%), while most of the bacterial sequences retrieved belonged to *Proteobacteria* (76.71%). Three bacterial strains isolated from decaying OPEFB, designated as S18, S20, and S36, appeared to grow with extracted OPEFB-lignin and Kraft lignin (KL) as the sole carbon source. 16S rRNA gene sequencing identified the 3 isolates as *Paenibacillus* sp.. The molecular weight distribution of KL before and after degradation showed significant depolymerization when treated with bacterial strains S18, S20, and S36. The presence of low-molecular-weight lignin-related compounds, such as vanillin and 2-methoxyphenol derivatives, which were detected by a GC-MS analysis, confirmed the KL-degrading activities of isolated *Paenibacillus* strains.

The depletion of fossil fuel reserves and the impact of climate changes induced by the emission of greenhouse gases from fossil fuels have recently increased the need for renewable and sustainable energy sources (9). Malaysia, a developing country with significant dependency on fossil fuels, has taken early precautions by shifting to renewable energy sources in order to ensure a sustainable and secure energy supply (2). The lignocellulosic biomass, which often refers to plants or plant-based materials, is an alternative source with the greatest potential to replace fossil fuels because it is abundant, inexpensive, and economically and environmentally friendly (9). Plantation residue, forestry waste, and agricultural residue are abundant biomass resources widely available in Malaysia (37). Oil palm is a more abundant source of plantation residue than rubber, cocoa, wood and timber, and pepper in Malaysia, in which the plantation area has increased from 54,000 hectares in 1960 to 5.81 million hectares in 2017, making Malaysia the second largest producer and exporter of palm oil after Indonesia worldwide (Malaysia Palm Oil Board. 2017. http://www.mpob.gov.my/). Oil palm (*Elaeis guineensis*) originating from the West Africa thrives in Malaysia’s tropical and humid climate (4). A long economic life span in combination with governmental policies and initiatives to support the sustainable production of palm oil are among the key factors responsible for the massive production of this crop in Malaysia (40).

Parallel to this gigantic growth of the palm oil industry, a vast amount of oil palm biomass is generated every year. Oil palm empty fruit bunches (OPEFB) are the main solid waste from the palm oil milling process and traditionally are incinerated and recycled into the plantation as fertilizer or used as soil mulch (Malaysia Palm Oil Board. 2017. http://www.mpob.gov.my/). However, these practices may create environmental pollution because incineration releases gases with particulates, such as tar, and indiscriminate dumping may cause the additional emission of methane into the atmosphere (41). Since the OPEFB biomass primarily consists of lignocellulosic materials, such as cellulose (57.8%), hemicellulose (21.2%), and lignin (22.8%) (38), efforts to manage these waste products by converting them into value-added products, including bio-fuels and biopolymer production components, have been attracting increasing interest among researchers (31). Briefly, cellulose and hemicellulose are polysaccharides that are easily hydrolyzed into fermentable sugars, whereas lignin is a complex heteropolymer composed of non-fermentable phenylpropane units linked by various types of C-C and C-O-C bonds that make it difficult to break down (35). The complex structure of lignin is the major obstacle to the utilization of the OPEFB biomass as a feedstock for carbon-based fuels, chemicals, and biomaterial production. Despite the natural recalcitrance of lignocellulose, several microorganisms, including fungi and bacteria, are known to be involved in lignocellulose degradation (7). Microbes are common inhabitants of decaying plants and wood. Decaying and decomposition processes by decomposer organisms are complex and principally important in forest biodiversity, nutrient cycling, and the carbon balance (27). Microbes commonly decompose most plant cell wall polymers into simpler compounds via enzymatic activities under aerobic or anaerobic conditions (27). Some microorganisms may produce enzymes that simultaneously or selectively degrade lignocellulose.

Fungi are the predominant decomposers of wood decay, and have the ability to produce a series of extracellular lignocellulolytic enzymes, including lignin peroxidases, Mn peroxidases, versatile peroxidases, and laccase- or laccase-like multicopper oxidases (LMCO) (32). White-rot *Basidiomycetes* (*e.g. Phanerochaete chrysosporium*) are the most studied and well-known fungi that degrade all the structural components of wood (cellulose, hemicellulose, and lignin), either simultaneously or sequentially, whereas brown-rot *Basidiomycetes* (*e.g. Fomitopsis palustris*) degrade cellulose and hemicellulose and oxidize lignin via a mechanism that relies on hydroxyl radicals (11, 18). A number of bacteria, including *Actinobacteria*, *Alphaproteobacteria*, and *Gammaproteobacteria*, have also been reported to oxidize lignin (7). However, limited information is currently available on the bacterial enzymes involved in the breakdown of lignin. Bacteria generally play a main role in the mineralization of large amounts of lignin-derived low-molecular-weight compounds in soils (23). The bacterial ability to use low-molecular-weight lignin indicates that bacteria have many unique and specific enzymes that catalyze the production of various useful compounds (23). For example, *Streptomyces viridosporus* T7A has been shown to degrade lignin by an extracellular lignin-inducible peroxidase enzyme (30) and *Sphingobium* sp. SYK-6 degrades dimeric lignin compounds via the protocatechuate (PCA) 4,5-cleavage pathway or multiple 3-O-methylgallate (3MGA) catabolic pathways (23). Several other known ligninolytic bacteria are *Pseudomonas putida* mt-2, *Rhodococcus jostii* RHA1, *Nocardia* sp., *Arthrobacter* sp., *Comamonas* sp., *Acinetobacter* sp., *Burkholderia* sp., and *Thermobifida fusca* (7). Bacterial enzymes are considered to be more cost-efficient than fungal enzymes due to multi-enzyme production with increased functionality and higher specificity as well as higher tolerance towards diverse forms of environmental stress. Furthermore, fungal enzymes, which are often less robust in terms of thermal and pH stabilities, may be costly to produce and difficult to optimize via genetic and protein engineering (7).

In the present study, the microbial diversity of decaying OPEFB was identified using Illumina MiSeq sequencing. Potential lignin-degrading bacteria were isolated from decaying OPEFB using extracted OPEFB-lignin and Kraft lignin (KL) as their sole carbon source. A thorough examination of microbial diversity and potential lignin-degrading bacteria from decaying OPEFB may lead to the discovery of novel ligninolytic enzymes and activities; these may then be useful in the development of new technologies for the production of biofuels and other value-added products from biomass resources.

## Materials and Methods

### Sampling

Fresh OPEFB and decaying OPEFB, which were left for more than six months in plantation, were collected from the Malaysia Palm Oil Board (Labu Sime Darby Plantation, Negeri Sembilan, Malaysia). Fresh and decaying OPEFB were cut into small pieces and stored at 4°C before being used.

### DNA extraction and preparation of the library for Illumina MiSeq sequencing

Total DNA was isolated from decaying OPEFB samples by the bead-beating method using the Mo Bio Powersoil DNA Isolation Kit (MO BIO Laboratories, Carlsbad, CA, USA) according to the manufacturer’s instructions. Briefly, approximately 0.25 g of each decaying OPEFB sample was measured and added to a powerbead tube, and this was followed by steps for homogenization, cell lysis, precipitation, and the removal of all non-DNA substances and traces that may interfere with DNA purity or the polymerase chain reaction (PCR). The quality and concentration of DNA in samples were assessed using the Qubit^®^ 2.0 DNA kit (Life Technologies, Carlsbad, CA, USA). Extracted DNA samples were sent to Sangon Biotech (Shanghai, China) for PCR amplification and sequencing. Bacterial diversity was evaluated by sequencing the V3 region of the 16S rRNA gene using the primers 341F (5′CCTACGGGNGGCW GCAG3′) and 805R (5′GACTACHVGGGTATCTAATCC3′), while fungal diversity was evaluated by sequencing the V4 region of the 18S rRNA gene using the primers 18SV4F (5′GGCAAGTCTGG TGCCAG3′) and 18SV4R (5′ACGGTATCTRATCRTCTTCG3′). The Sangon agarose recovery kit was used to recover DNA products, which were further quantified using the Qubit^®^ 2.0 DNA kit. DNA samples were then ready for library preparation and sequencing.

### Bioinformatics and data analysis

Raw sequencing reads were quality-filtered and analyzed using FLASH software (v1.2.7). Sequences were extracted based on 100% barcode similarity. The extraterritorial sequences of target areas and chimeras were removed using PRINSEQ software (PRINSEQ-lite 0.19.5). Reads were then clustered into OTUs at 97% sequence similarity using UCLUST software (UCLUST v1.1.579). Representative sequences were submitted to the Ribosomal Database Project classifier, which is based on Bergey’s taxonomy, for taxonomic unit classification. Alpha diversity was evaluated by richness rarefaction curves, the Shannon index, Chao1 index, and ACE index and coverage.

### Preparation of extracted OPEFB-lignin and FTIR analysis

The procedure for lignin extraction from the biomass was modified according to Sainsbury *et al*. (34). Fresh samples of OPEFB collected from oil palm mills were ground into fine pieces. Approximately 15 g of grounded OPEFB was mixed with 1.5% w/v NaOH (300 mL) at room temperature for 2 h. The resulting slurry was filtered and acidified to pH 6.5 with acetic acid. The total filtrate volume was then concentrated in the oven at 60°C for 24 h. The concentrated filtrate was then mixed with 5 volumes of ethanol and left overnight. Precipitated hemicellulose was removed by filtration. The filtrate was then acidified to pH 1.5 with 6 M of HCl and left for 24 h. The lignin precipitate was collected by centrifugation at 10,000×*g* for 10–15 min, and pellets were frozen overnight. Approximately 200 mg of OPEFB-lignin was obtained and stored at 4°C until further use.

Extracted OPEFB-lignin and commercial KL were analyzed using a Fourier Transform Infrared (FTIR) spectrometer (Perkin Elmer, Waltham, MA, USA) in the solid and liquid phases. In the solid form, lignin was analyzed as a solid disc when mixed with KBr and compressed into a pellet. In the liquid phase, 0.25 g of lignin was diluted in 10 mL of 1N NaOH as the solvent. The lignin solution was analyzed using the Attenuated Total Reflection (ATR) technique. All peaks in the spectrum were collected and major bands were elucidated. FTIR spectra for lignin were compared and analyzed.

### Medium composition and isolation of bacteria

Approximately 1.0 g of decaying OPEFB was cut and mixed with 1 mL of 0.7% NaCl for a stock solution, of which 0.1 mL was serially diluted until a dilution of 10^4^ was reached with 0.9 mL of 0.7% NaCl. Serially diluted solutions were cultured on selectively modified W-minimal medium plates, adapted from Seto *et al*. (36). A total of 2.5 g L^−1^ (0.25%) of KL or OPEFB-lignin solution was filter-sterilized with the addition of NaOH. In the preparation of 200 mL of W-minimal medium with 1.5% agar, 20 mL of filter-sterilized KL or OPEFB-lignin was used as the sole carbon source, and 1 mL of cycloheximide was added to inhibit fungal growth. One hundred microliters of each serially diluted solution was spread on a W-minimal OPEFB-lignin agar plate and incubated at 30°C for 7 d until colonies developed. Each colony was then purified on new W-minimal KL agar medium using the streak plate technique.

### 16S rRNA gene sequencing

Regarding DNA extraction, cells for each strain were collected from agar plates and mixed with 100 μL of autoclaved water. The cell solution was disintegrated with an ultrasonicator (Amp 3.0, cycle 0.5) 5 times. One hundred microliters of phenol-chloroform was added and mixed by tapping. The cell solution was then centrifuged at 10,000×*g* for 3 min. The upper layer of DNA was collected and stored at 4°C for further analyses. PCR was performed with GoTaq^®^ DNA polymerase (Promega, Madison, WI, USA) according to the manufacturer’s instructions under the following conditions: 30 cycles of denaturation at 95°C (30 s), annealing at 55°C (30 s), and extension at 72°C (1.3 min). Gene fragments of isolated cultures were amplified using the forward primer 27F (5′AGAGTTTGATC MTGGCTCAG3′) and reverse primer 1492R (5′TACGGYTACCT TGTTACGACTT3′). The amplification lengths of PCR products were then visualized by gel electrophoresis. Approximately 1.5-kb fragments of amplicons were sequenced and analyzed using the Basic Local Alignment Search Tool (BLAST) algorithm on the NCBI web server (http://blast.ncbi.nlm.nih.gov/) and Clustal Omega web server (https://www.ebi.ac.uk/Tools/msa/clustalo/). Sequences were deposited in the GenBank database (http://www.ncbi.nlm.nih.gov/). A phylogenetic tree was then constructed using the neighborjoining method with MEGA 5.0 software.

### GPC analysis

The molecular weight distribution of KL was investigated using aqueous gel permeation chromatography (GPC) (LC-20AD GPC, Shimadzu, Kyoto, Japan). A total of 0.25% of KL was incubated in W-minimal medium degraded by bacterial strains S18, S20, and S36 at 30°C for 7 d. A sample without the inoculating bacteria was analyzed as the control. A KL sample (10 mg) was weighed and 2.3 mL of glacial (anhydrous) acetic acid was added. The reaction mixture was stirred for time periods ranging between 15 min and 20 h, and 0.25 mL (3.38 mmol) of acetyl bromide was then added. The reaction mixture was finally stirred at room temperature for 20 h. Acetic acid and excess acetyl bromide were evaporated with a rotary evaporator, followed by high vacuum drying at 25–30°C for 30–45 min (3). Dried acetylated samples were dissolved in tetrahydrofuran (THF) and then filtered (0.22-μm nylon membrane syringe filters) before the GPC analysis. THF (HPLC grade, without stabilizer) was used as the mobile phase with a flow rate of 0.5 mL min^−1^. The columns used were 600 mm×4.6 mm i.d., Styragel HR-5E and Styragel HR-1, connected in series with a 30 mm×4.6 mm i.d. guard column of the same material (Waters, Milford, MA, USA). The system was calibrated with polystyrene standards (533, 953, 2,620, 16,200, and 193,000 Da) using UV detection at 280 nm. Retention times were converted into molecular weights by applying a calibration curve established using polystyrene standards.

### Metabolite characterization by the GC-MS analysis

In the degradation study, each purified strain was inoculated in Luria Broth medium and incubated until the optimal density (OD) at 600 nm reached approximately 1.0. Fifty milliliters of the cell cultures was aseptically transferred into a 50-mL conical flask and cells were harvested by centrifugation at 10,000×*g* for 10 min. Pelletized cells were re-suspended in 100 mL of W-minimal medium in the conical flask twice prior to washing and then inoculated with 10 mL of W-minimal medium with 0.25% of KL at 30°C for 7 d while shaking continuously at 160 rpm. One-milliliter aliquots of each culture were withdrawn periodically at 24-h intervals. One-milliliter aliquots of each sample were centrifuged at 10,000×*g* at 4°C for 10 min. The supernatant was collected and transferred to a new tube. Five hundred microliters of the supernatant was acidified to pH 1–2 with 1 M of HCl, 0.2 g of NaCl and 500 μL of ethyl acetate were then added, and the supernatant was centrifuged at 10,000×*g* at 4°C for 10 min. The organic layer was separated, and the upper layer was mixed with 0.2 g of Na_2_SO_4_. After centrifugation at 10,000×*g* at 4°C for 10 min, 100 μL of the upper layer was collected and stored at −20°C for the GC-MS analysis. One microliter of silylated compounds was injected into the GC–MS system (Shimadzu). Helium was used as the carrier gas (0.7 bar) at a flow rate of 1.0 mL min^−1^ through a fused silica SH-Rxi-5Sil MS capillary column (30 m×0.25 mm i.d., 0.25 μm film thickness). The splitter injector was maintained at 300°C on the split mode and column temperature was programmed as follows: 100°C held for 5 min and then increased by 10°C min^−1^ until 300°C, where it was held for 5 min. Ion source temperature was maintained at 200°C. In the full-scan mode, electron ionization mass spectra in the range of 30–500 (m/z) were recorded. The identification of low-molecular-weight compounds derived from lignin-degrading bacteria was performed by comparing mass spectra to the National Institute of Standards and Technology 11 (NIST 11) library, which is available in the instrument.

### Data deposit

Illumina MiSeq sequencing data and nucleotide sequences identified in the present study were deposited in the NCBI database with the accession numbers SRP076826 and KY305299, KY305300, and KY305301, respectively.

## Results

### Fungal and bacterial diversities in decaying OPEFB

In order to identify the microbial community involved in lignocellulosic degradation, fungal and bacterial diversities were both characterized by sequencing the V4 region of the 18S rRNA gene and V3 region of the 16S rRNA gene of microbes from decaying OPEFB, respectively. Alpha diversity was assessed by calculating the Shannon diversity index, ACE index, and the Chao1 richness index based on OTUs (Operational Taxonomic Unit) with 97% identity. Following the quality filtering process and removal of chimeric sequences, 16,420 reads from fungal sequences and 20,856 reads from bacterial sequences with an average sequence length of 450 bp were recovered. Based on the α-diversity index, at 97% similarity, the numbers of fungal and bacterial OTUs were 5,982 and 7,963, respectively. In the present study, the major fungal phylum detected in decaying OPEFB was unknown (83.33%), whereas the most prominent fungal phylum detected from decaying OPEFB was *Ascomycota* (14.43%), followed by the phyla *Glomeromycota* (1.56%), *Chytridiomycota* (0.30%), and *Basidiomycota* (0.30%), and the subphylum *Mucoromycotina* (0.09%) (Table S1A). *Sordariomycetes* (7.60%), *Eurotiomycetes* (4.33%), *Saccharomycetes* (2.30%), and *Glomeromycetes* (1.56%) were the most commonly detected fungal classes in the sample, while the most abundant known fungal genera included *Sarocladium* (3.11%), *Emericella* (2.81%), *Verticillum* (2.35%), *Candida* (1.83%), *Aspergillus* (1.22%), *Rhizophagus* (1.13%), and *Sordaria* (0.71%) (Table S1A). Regarding bacterial diversity, the most dominant phylum recovered from decaying OPEFB samples was *Proteobacteria* (76.71%), followed by the phyla *Bacteroidetes* (8.26%), *Actinobacteria* (5.60%), *Firmicutes* (4.79%), and *Acidobacteria* (2.03%) (Table S1B). The most abundant bacterial classes were dominated by *Alphaproteobacteria* (57.38%), followed by *Gammaproteobacteria* (10.16%), *Betaproteobacteria* (9.06%), *Sphingobacteria* (6.82%), and *Actinobacteria* (5.60%). Furthermore, the most abundant bacterial genera were dominated by *Sphingomonas* (13.96%), *Mesorhizobium* (8.44%), *Shinella* (7.89%), and *Luteimonas* (6.09%) (Table S1B).

### Isolation and identification of potential OPEFB lignin-degrading bacteria

In the isolation of potential lignin-degrading prokaryotes from decaying OPEFB, W-minimal medium with 0.25% of extracted OPEFB-lignin was used to obtain candidates for the degradation of specific oil palm lignin. Based on FTIR results (Fig. S1), extracted-OPEFB lignin and commercial KL both shared similar features with the presence of similarly shaped peaks. A broad peak was identified in both lignins within the range of 3,200 to 3,400 cm^−1^, indicating the presence of a hydroxyl group (OH) from extracted-OPEFB lignin and commercial KL. In addition, they shared almost similar peak values of 1,641 cm^−1^ in KL and 1,642 cm^−1^ in OPEFB lignin, which may be attributed to the presence of C=C stretching. Moreover, the peak value at 676 cm^−1^ in OPEFB lignin, and at 602 cm^−1^ and 666 cm^−1^ in KL showed the presence of C-H bending in both lignins. Sixteen bacterial strains grew well on W-minimal medium-extracted OPEFB-lignin agar plates after 7 d of incubation at 30°C. Sixteen bacterial strains were further plated on W-minimal medium containing 0.25% of KL as the sole carbon source at 30°C for 7 d (Fig. S2). Among the 16 isolates, 3 bacterial strains that morphologically showed clear white spores and leathery formation, which are similar to spore-forming *Streptomyces* sp., were selected. The 3 potential lignin-degrading bacteria were designated as S18, S20, and S36. These 3 bacterial strains were plated on W-minimal medium without KL; however, no bacterial growth was observed on the plate, suggesting that bacterial strains S18, S20, and S36 grew with KL as their carbon source. Based on the BLAST analysis of 16S rRNA gene sequencing, the bacterial strains S18, S20, and S36 showed 91, 97, and 98% sequence similarities with the *Paenibacillus lautus* strain NRRL NRS-666, strain AB236d, strain NBRC 15380, and strain JCM 9073, respectively (Fig. S3). Bacterial identification was further analyzed using a Clustal Omega analysis and these 3 bacterial strains showed high similarities to known 16S rRNA gene sequences from *P. lautus*.

### Evaluation of lignin-degrading activities by isolates

**Fig. 1:**
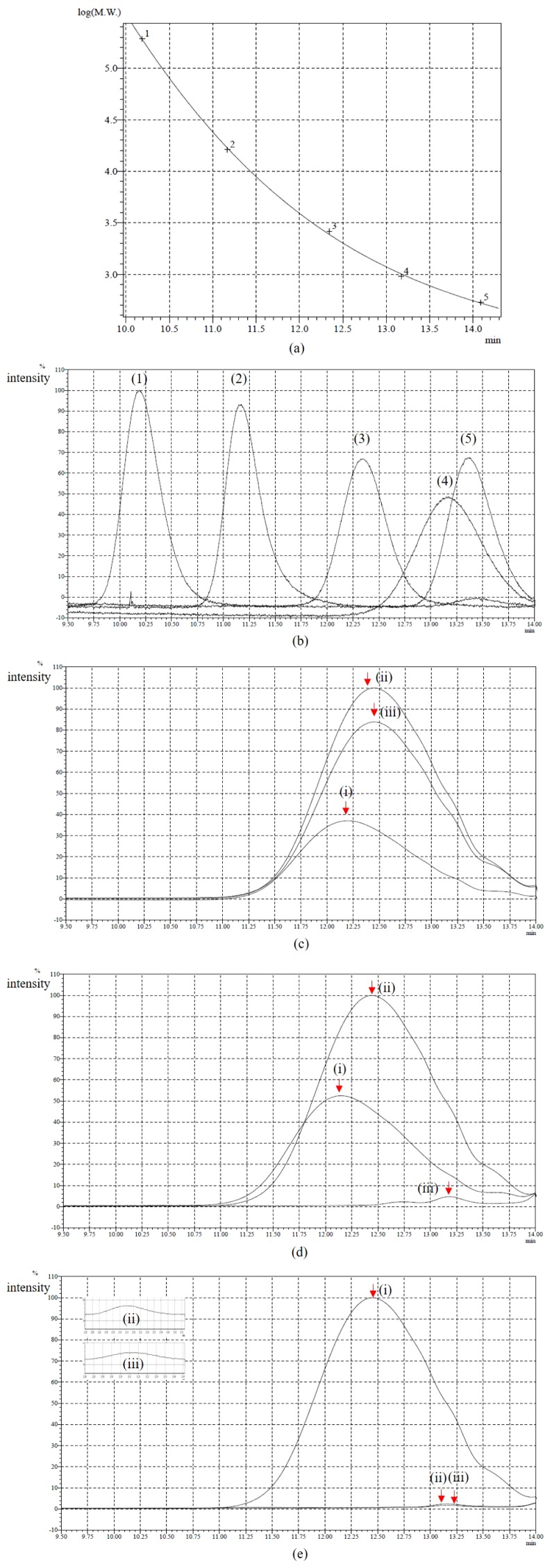
GPC chromatogram data. a) Calibration line for GPC standard markers. b) Molecular weights of the standard markers: (1): 193 000 Da, (2): 16 200 Da, (3): 2 620 Da, (4): 953 Da, (5): 533 Da, c) S18, d) S20, e) S36, (i): day 0, (ii): day 4, (iii): day 7

**Fig. 2:**
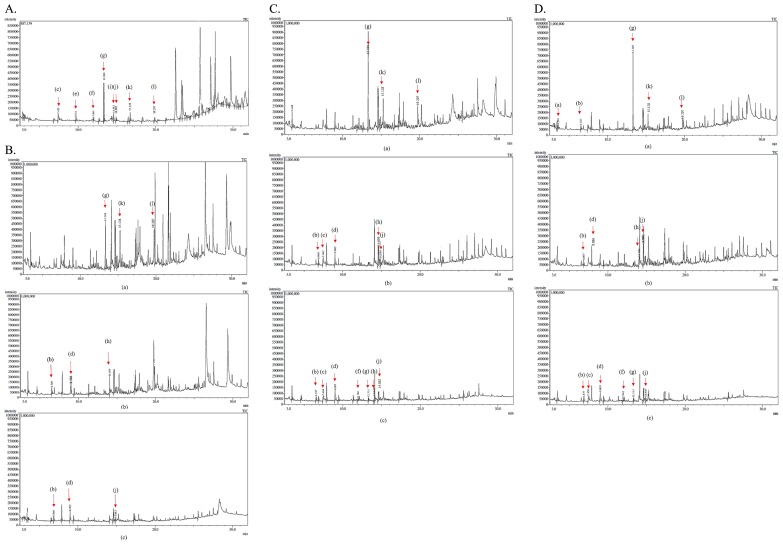
A: TIC of metabolites produced by control (untreated samples). B: TIC of metabolites produced by S18 from KL degradation: a) day 0, b) day 4, and c) day 7. C: TIC of metabolites produced by S20 from KL degradation on a) day 0, b) day 4, and c) day 7. D: TIC of metabolites produced by S36 from KL degradation on a) day 0, b) day 4, and c) day 7. Peaks of the corresponding retention times: (a) 1,2-ethanediol, (b) 2,3-butanediol, (c) 2-methoxy phenol, (d) benzoic acid, (e) catechol, (f) 2-methoxy-4-vinylphenol, (g) vanillin, (h) 4-(hydroxymethyl)-2-methoxyphenol, (i) apocynin, (j) 3,5-di-tert-butylphenol, (k) ethyl 4-ethoxybenzoate, (l) di-isobutyl phthalate.

GPC was used to measure the molecular weight distribution of KL on days 0, 4, and 7 of the treatment with bacterial strains S18, S20, and S36. Based on the results obtained (Fig. 1), KL samples treated with S18 were not completely depolymerized on day 7 because molecular weights remained high. However, a broadening decrease in intensity and a shift to a longer retention time of the peak were observed with S20 and S36, suggesting that KL samples were almost completely depolymerized after 7 d of incubation. Meanwhile, the degradation activities of bacterial strains were confirmed by the GC-MS analysis, which was proven to be a very suitable method for analyzing low-molecular-weight compounds released from lignin degradation (29). Regarding total ion chromatograph (TIC) patterns corresponding to the compounds extracted with ethyl acetate from the untreated sample (control) and treated samples, S18, S20, and S36 were shown in Fig. 2A, B, C, and D and the peak identity was depicted in Table 1. The number of low-molecular-weight compounds detected from the control (the untreated sample) revealed that the partial degradation of KL occurred during the industrial production process (39). Some of the compounds, including catechol, apocynin, vanillin, and di-isobutyl phthalate, disappeared after 7 d of incubation, suggesting that these compounds were further degraded. A few new compounds, such as benzoic acid and 2,3-butanediol, 4-(hydroxymethyl)-2-methoxyphenol, were detected on days 4 and 7 of the incubation, indicating the ability of isolates to depolymerize KL.

## Discussion

### Fungal and bacterial diversities in decaying OPEFB

OPEFB, which is left over a period at palm oil plantation after the removal of sterilized fruit, undergoes decomposition or decay. Malaysia has a year-round highly humid and tropical climate that may influence many microbial diversities, including fungi and bacteria as the main decomposers in decaying OPEFB. Previous studies reported a higher number of known fungal phyla, such as *Basidiomycota* and *Ascomycota*. Tian *et al*. (42) showed that the most abundant fungal phylum found in soil in Xishuangbanna, a tropical forest in China, was *Mucoromycotina* followed by unclassified *Zygomycota*, *Ascomycota*, and *Basidiomycota*, whereas *Ascomycota* and *Basidiomycota* were the most diverse and abundant phyla detected in rainforest soils of the western Amazon basin (28). Toju *et al*. (43) also reported that *Ascomycota* was the most abundant phylum detected in a subtropical secondary forest located on Yakushima Island, Japan. In contrast, our results differ from previous findings because the percentage of unknown fungal phyla was higher than known fungal phyla. In a study by Buée *et al*. (6) that used 454 sequencing to measure fungal diversity in forest soils, 71.5% of their sequences lacked explicit taxonomic annotation. Hibbett *et al*. (16) reported that the vast majority (>95%) of fungal diversity remains undetected and much of the detected fraction lacks scientific names. In addition, environmental sequencing mostly has ‘fungal-specific’ primers that have been designed based on known sequences and are often biased towards the *Dikarya* subkingdom (*Ascomycota* and *Basidiomycota*) (5). Therefore, to some extent, poorly-known fungi are constrained to remain largely unknown, and although molecular data, mainly generated by next generation sequencing technologies, offer an important insight into fungal diversity, the true power of this approach will only be revealed in time when sampling coverage becomes sufficiently large and more level taxa will be detected and described in the future (21). Therefore, we consider unique degradation mechanisms to be present in decaying OPEFB in the Tropics that may have resulted in the abundant existence of unknown fungi, and these diverse fungal lineages have yet to be characterized. *Ascomycota*, which is reportedly the largest fungal phylum and one of the most diverse and ubiquitous phyla of eukaryotes, showed the highest percentage among the known phyla in our decaying OPEFB. Although previous studies demonstrated that *Ascomycetes* degrade cellulose and hemicellulose, some also showed that *Ascomycetes* are involved in lignin degradation through the secretion of laccases and lignin peroxidases (12). The fungal phylum *Basidiomycota* in decaying OPEFB samples was previously reported to be involved in the late stages of decomposition of decaying plants or wood, with the inner tough structure being broken down (42, 46). The fungal phyla *Glomeromycota* and *Chytridiomycota* were also retrieved in our decaying OPEFB samples. However, limited information is currently available on *Glomeromycota* and *Chytridiomycota*, making them more unique than *Ascomycota* and *Basidiomycota*.

Although fungi are the dominant agents of wood decomposition, bacteria are known to inhabit decaying wood, but have not been examined to the same extent as fungi in this habitat (20). Previous studies showed that *Proteobacteria*, *Acidobacteria*, and *Actinobacteria* were the dominant taxonomic groups in decaying deadwood logs (17), forest soils (26), and decaying wood samples (44), while a study by Ventorino *et al*. (45) demonstrated that the phyla *Actinobacteria*, *Proteobacteria*, *Bacteroidetes*, and *Firmicutes* were the most abundant taxa found in different lignocellulosic vegetable biomasses. Based on the present results, the most dominant phylum was *Proteobacteria*, followed by *Bacteroidetes*, *Actinobacteria*, *Firmicutes*, and *Acidobacteria*. In contrast to previous findings, we suggest that although the types of phyla are similar, the pattern of taxa abundancy differs for each sample or respective habitat. *Proteobacteria*, comprising the vast majority of known Gram-negative bacteria, was the most abundant phylum recovered from decaying OPEFB. The large abundancy of *Proteobacteria* has been associated with a high availability of carbon (13) and members of the phylum were previously characterized as biomass degraders. *Bacteroidetes* represent the second most abundant phylum within decaying OPEFB and are degraders of polymeric organic matter, particularly that in the form of polysaccharides and proteins (10). Mhuantong *et al*. (25) also reported that the phylum *Bacteroidetes* produces the largest repertoire of carbohydrate-degrading enzymes, particularly cellulases, oligosaccharidedegrading enzymes, and endo-hemicellulases. Meanwhile, the phylum *Actinobacteria* is known to comprise active biomass degraders, and is expected to be among the initial colonizers of deadwood (17), and the phylum *Firmicutes* was found to be common in natural processes, such as rice straw compost and decaying wood (45). Based on the present results and previous findings, the majority of bacterial and fungal phyla may be involved in the degradation of cellulose, hemicellulose, and lignin due to the production of various lignocellulolytic and synergistically-acting enzymes with some degradation occurring in the early, intermediate, or late stages, thereby contributing to the decay of OPEFB lignocellulosic residues in nature. Further studies on these phylum enzymatic activities and the genes involved in the degradation of decaying OPEFB in the Tropics are needed in order to obtain more detailed information, which will be beneficial for the discovery of new microbial enzymes to improve the lignocellulosic biomass conversion process.

### Isolation and identification of potential OPEFB lignin-degrading bacteria

In the present study, the first screening of potential lignin-degrading bacteria from decaying OPEFB was performed based on their ability to utilize natural OPEFB lignin in W-minimal medium. To date, studies have used wheat straw lignin (1, 15, 47) and switchgrass (19, 22), but not OPEFB-derived lignin as the sole carbon source; therefore, the 16 bacterial isolates that grew well on W-minimal medium-extracted OPEFB-lignin agar plates are unique because they appear to exclusively use OPEFB-derived lignin as the sole carbon source for their growth. Due to the time-consuming extraction process and low yield amounts of extracted-OPEFB lignin gained, commercial KL was used in subsequent experiments. FTIR spectra showed that extracted-OPEFB lignin contained similar functional groups as KL, confirming that extracted-OPEFB lignin is similar enough to be replaced by KL in future studies.

Based on the present results, bacterial strains S18, S20, and S36 appeared to metabolize KL as their sole carbon source and, thus, the total amount of lignin degraded may be higher for these strains than those using lignin as a co-metabolite (39). We initially hypothesized that our spore-forming strain may be *Streptomyces*, which are good lignin degraders because they produce an extensive range of bioactive compounds, including lignocellulolytic enzymes (7, 33). However, our strains were identified as *Paenibacillus* sp., which is of interest because studies on the lignin-degrading activities of *Paenibacillus* strains remain limited. There are few studies with *Paenibacillus* on ligninolytic degradation, including *Paenibacillus* sp., isolated from pulp paper mill waste, which was only able to degrade lignin in the presence of glucose and peptone as a supplementary source of carbon and nitrogen (8). In contrast, *P. glucanolyticus* SLM1, isolated from pulp mill waste, degraded lignocellulose, lignin, and aromatic lignin-related compounds under aerobic and anaerobic conditions (24). Although there were few reports on *Paenibacillus* strains as lignin degrader, none were isolated from the OPEFB plantation. These strains were also shown to grow well on lignin with the support of a co-substrate, such as glucose. In contrast, to the best of our knowledge, *Paenibacillus* strains are the first lignin-degrading bacteria to be isolated from a relatively unexplored environment, the OPEFB biomass, which was collected from the oil palm plantation in a tropical country, Malaysia.

### Evaluation of lignin-degrading activities of isolates

The lignin-degrading activities of all 3 isolates were evaluated quantitively by GPC and qualitatively by a GC-MS analysis. Filley *et al*. (14) proposed that lignin degradation occurs via 3 main reactions: depolymerization, side chain oxidation, and demethylation. The most important aspects of lignin biodegradation are depolymerization and solubilization, which result from bond cleavage reactions by extracellular enzymes. Based on GPC results, bacterial strains S20 and S36 both achieved higher rates of degradation than strain S18 because treated KL was completely depolymerized on day 7. However, according to GC-MS results, all 3 isolates, S18, S20, and S36, were capable of degrading KL to LWM lignin-related compounds after 7 d of treatment. Some compounds, such as catechol, apocynin, ethyl 4-ethoxybenzoate, and di-isobutyl phthalate, were present in the control, but were not detected on day 4 or 7, suggesting that the isolates degraded these compounds. Most of the metabolites produced from the isolated strains were from guaiacyl-type lignins (G-type), such as vanillin, 2-methoxyphenol, 2-methoxy-4-vinylphenol, and 4-(hydroxymethyl)-2-methoxyphenol, suggesting that the bacterial isolates attacked the guaiacyl (G) unit of the lignin polymer. 2,3-Butanediol, a secondary product of the fermentative pathway from pyruvate, was also detected on days 4 and 7 for bacterial strains S18, S20, and S36. The compounds identified in bacterial-degraded samples provide an insight into the biochemical modification of KL into LWM lignin-related compounds. Nevertheless, differences in the patterns of lignin degradation by S18, S20, and S36 suggest variations in ligninolytic mechanisms among the strains; therefore, *Paenibacillus* is regarded as the main potential contributing prokaryote to wood lignin degradation in decaying OPEFB. Further studies on the identification of intermediate metabolites and enzymes using a genome sequence analysis are needed in the near future.

## Conclusion

Fungal and bacterial diversity data increase our knowledge on the diversity of lignocellulosic microorganisms in decaying OPEFB in the Tropics. Microorganisms are more diverse in the Tropics than in other temperate countries due to their adaptability to the tropical and humid climate. The study of the biodiversity of microorganisms from decaying OPEFB may provide beneficial information as there may be diversity of microorganisms’ enzymes and lignocellulose degradation systems. The discovery of novel lignin-degrading bacterial enzymes may provide important evidence to show that bacterial enzymes have the ability to degrade and/or modify the structure of recalcitrant lignin and release fermentable sugars from OPEFB lignocellulose. The results showing that *Paenibacillus* sp. are lignin degraders of OPEFB and KL are of interest. Only a limited number of studies have reported this species as a rare wood lignin degrader. Further studies are needed on the strain’s genes, enzymes, and degradation pathways in order to understand the fundamentals of wood lignin degradation by this particular strain. This information will also be advantageous because bacterial enzymes may facilitate or enhance the pre-treatment process in different lignocellulosic industries and, thus, improve OPEFB waste management in the Tropics of Southeast Asia.

## Supplementary Information



## Figures and Tables

**Table 1:** Chromatographic peak identification of metabolites produced by C (control), S18, S20, and S36 on days 0, 4, and 7 from KL degradation.

RT (min)	Identified compounds	C	Day 0			Day 4			Day 7		
		
			S18	S20	S36	S18	S20	S36	S18	S20	S36
3.857	1,2-ethanediol	−	−	−	+	−	−	−	−	−	−
6.540	2,3-butanediol	−	−	−	+	+	+	+	+	+	+
7.461	2-methoxyphenol	+	−	−	−	−	+	−	−	+	+
8.986	Benzoic acid	−	−	−	−	+	+	+	+	+	+
9.711	Catechol	+	−	−	−	−	−	−	−	−	−
11.957	2-methoxy-4-vinylphenol	+	−	−	−	−	−	−	−	+	+
13.301	Vanillin	+	+	+	+	−	−	−	−	+	+
14.039	4-(hydroxymethyl)-2-methoxyphenol	−	−	−	−	+	+	+	−	+	−
14.582	Apocynin	+	−	−	−	−	−	−	−	−	−
14.882	3,5-di-tert-butylphenol	+	−	−	−	−	+	+	+	+	+
15.128	Ethyl 4-ethoxybenzoate	+	+	+	+	−	−	−	−	−	−
19.207	Di-isobutyl phthalate	+	+	+	+	−	−	−	−	−	−

+; indicates presence, −; indicates absence
