# Supplementary material for: Microbial Diversity in Decaying Oil Palm Empty Fruit Bunches (OPEFB) and Isolation of Lignin-degrading Bacteria from a Tropical Environment
Source: Microbes Environ. 2019 Apr 23;34(2):161–8. doi: 10.1264/jsme2.ME18117 (PMC6594733; doi:10.1264/jsme2.ME18117)
Supplement: Supplementary file 1 [file 34_161_s1.pdf]

Supplemental Figure legends

Figure S1, FTIR spectra in liquid form (dissolve with NaOH). A: Extracted OPEFB lignin, B: Kraft lignin.

Figure S2, sixteen lignin degrading bacteria isolates on WM-KL agar plate; a) S1/S2, b) S3/S5, c) S7/S9, d) S11/S13, e) S16/S18, f) S20/S22, g) S23/S29, h) S32/S36

Figure S3, bacterial strains S18, S20, and S36 showed sequence similarities with *Paenibacillus lautus* 1. NR_115599.1, *Paenibacillus lautus* strain NRRL NRS-666, 4. NR_117185.1, *Paenibacillus lautus* strain AB236d, 5. NR_112724.1, *Paenibacillus lautus* strain NBRC 15380, and 7. NR_040882.1, *Paenibacillus lautus* strain JCM 9073, respectively.

A.

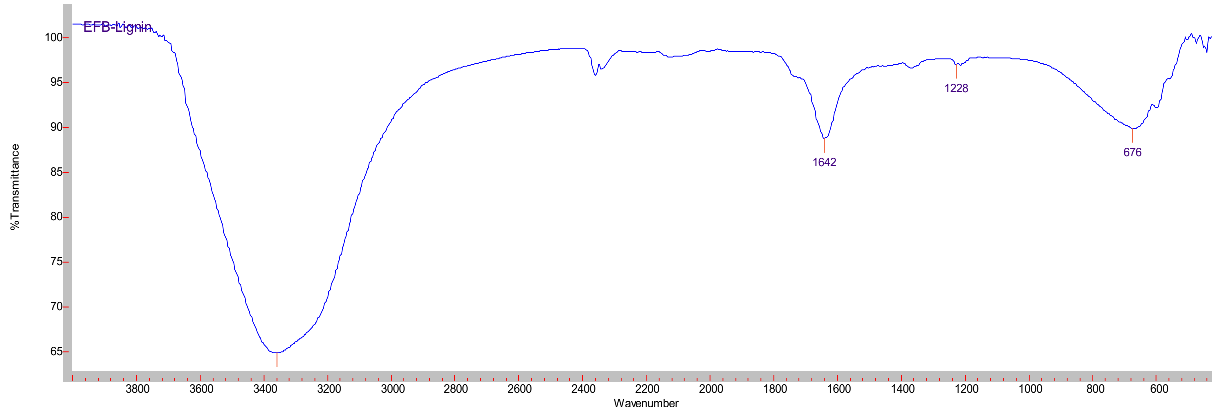

B.

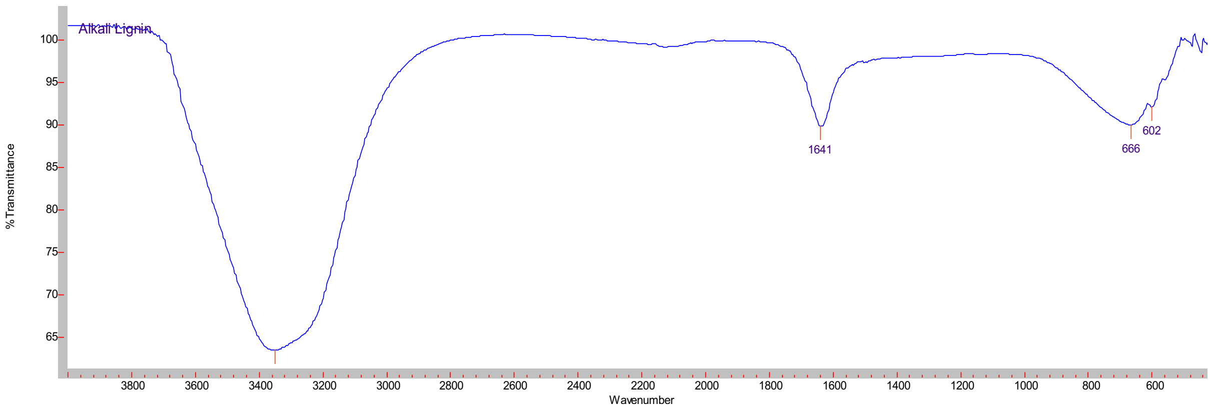

Supplemental Figure S1, Analhuda Abdullah Tahir et al.,

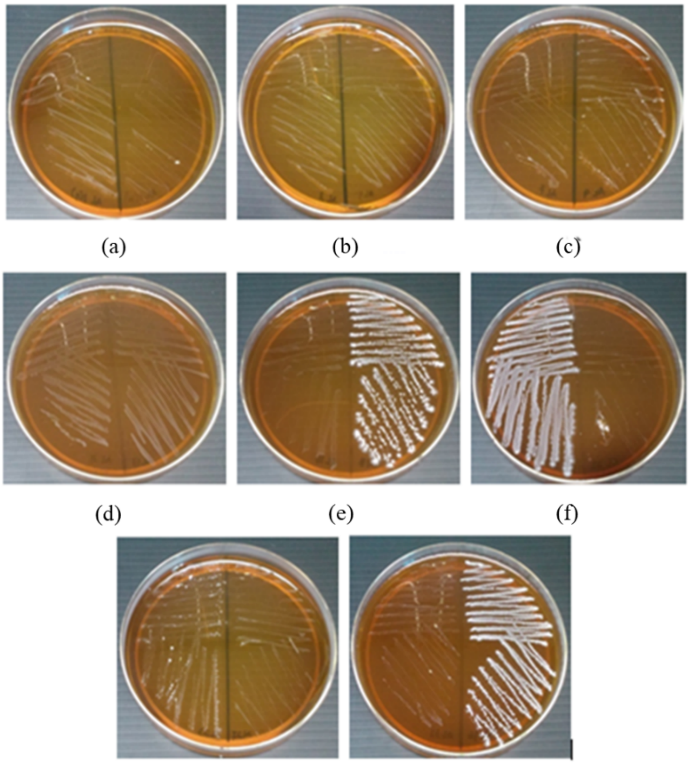

(a) (b) (c)

(d) (e) (f)

(g) (h)

Supplemental Figure S2, Analhuda Abdullah Tahir et al.,

Percent Identity Matrix - created by Clustal2.1

1: NR_115599.1	100.00	99.28	99.41	98.73	98.94	99.14	99.01
2: s36	99.28	100.00	100.00	100.00	99.82	99.82	99.82
3: s18	99.41	100.00	100.00	100.00	99.88	99.88	99.88
4: NR_117185.1	98.73	100.00	100.00	100.00	99.37	99.57	99.37
5: NR_112724.1	98.94	99.82	99.88	99.37	100.00	99.91	99.93
6: s20	99.14	99.82	99.88	99.57	99.91	100.00	100.00
7: NR_040882.1	99.01	99.82	99.88	99.37	99.93	100.00	100.00

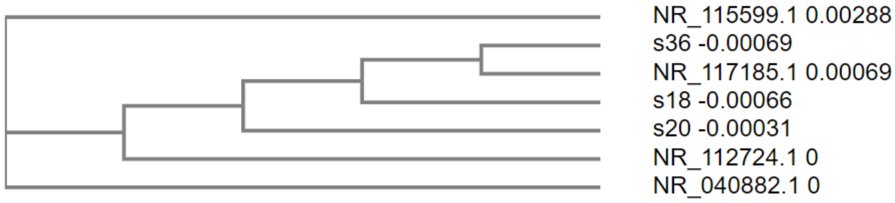

Supplemental Figure S3, Analhuda Abdullah Tahir et al.,

Table S1, A: Classification of different phyla, classes and genera for fungal diversity, B: Classification of different phyla, classes and genera for bacterial diversity. Others represent other genera or classes under each phylum.

A.

Phylum (%)		Class (%)		Genus (%)	
Unknown	83.33	Unknown	83.33	Unknown	83.33
Ascomycota	14.43	*Sordariomycetes*	7.60	*Sarocladium*	3.11
				Verticillium	2.35
				Sordaria	0.71
				Myrothecium	0.31
				Nectria	0.24
				Plectosphaerella	0.23
				Others	0.65
		Eurotiomycetes	4.33	*Aspergillus*	1.22
				Emericella	2.81
				Talaromyces	0.18
				Others	0.12
		Saccharomycetes	2.30	*Candida*	1.83
				Meyerozyma	0.12
				Others	0.35
		Pezizomycetes	0.14	*Ascobolus*	0.10
				Others	0.04
		Dothideomycetes	0.06	*Cladosporium*	0.05
				Others	0.01
Glomeromycota	1.56	*Glomeromycetes*	1.56	*Rhizophagus*	1.13
				Funneliformis	0.19
				Glomus	0.18
				Others	0.06
Chytridiomycota	0.30	*Chytridiomycetes*	0.30	*Blyttiomyces*	0.08
				Powellomyces	0.06
				Others	0.16
Basidiomycota	0.30	*Agaricomycetes*	0.30	*Coprinopsis*	0.19
				Others	0.11
Mucoromycotina	0.09	*Zygomycete*	0.09	*Lichtheimia*	0.05
				Others	0.04

B.

Phylum (%)		Class (%)		Genus (%)	
Proteobacteria	76.71	*Alphaproteobacteria*	57.38	*Sphingomonas*	13.96
				Mesorhizobium	8.44
				Devosia	4.62
				Porphyrobacter	2.93
				Rhizobium	2.78
				Ensifer	2.46
				Sphingosinicella	2.34
				Bosea	2.22
				Sphingobium	1.83
				Hoeflea	1.79
				Paracoccus	1.25
				Aminobacter	1.13
				Altererythrobacter	1.01
				Others	10.62
		Gammaproteobacteria	10.16	*Luteimonas*	6.09
				Succinivibrio	1.50
				Acinetobacter	0.37
				Ruminobacter	0.24
				Others	1.96
		Betaproteobacteria	9.06	*Shinella*	7.89
				Pseudorhodoferax	0.48
				Others	0.69
		Deltaproteobacteria	0.11	*Byssovorax*	0.06
				Others	0.05
Bacteroidetes	8.26	*Sphingobacteria*	6.82	*Olivibacter*	4.6
				Parapedobacter	0.90
				Flavihumibacter	0.62
				Others	0.70
		Bacteroidia	0.81	*Prevotella*	0.42
				Barnesiella	0.20
				Others	0.19
		Flavobacteria	0.34	*Flavobacterium*	0.26
				Others	0.08
		Bacteroidetes	0.29	*Ohtaekwangia*	0.24
				Prolixibacter	0.05

Actinobacteria	5.60	*Actinobacteria*	5.6	*Gordonia*	1.02
				Oerskovia	0.64
				Ilumatobacter	0.22
				Others	3.72
Firmicutes	4.79	*Bacilli*	3.12	*Bacillus*	1.16
				Others	1.96
		Clostridia	1.55	*Lachnospiracea*	0.27
				Ruminococcus	0.13
				Others	1.15
		Negativicutes	0.09	*Phascolarctobacterium*	0.06
				Anaerovibrio	0.015
				Selenomonas	0.015
		Erysipelotrichia	0.03	*Bulleidia*	0.015
				Holdemania	0.015
Acidobacteria	2.03	*Acidobacteria_Gp17*	0.03	*Gp17*	0.03
		Acidobacteria_Gp3	1.24	*Bryobacter*	0.32
				Gp3	0.92
		Acidobacteria_Gp4	0.74	*Gp4*	0.74
		Acidobacteria_Gp6	0.02	*Gp6*	0.02
Verrucomicrobia	1.41	*Verrucomicrobiae*	1.2	*Luteolibacter*	1.15
				Verrucomicrobium	0.05
		Spartobacteria	0.16	*Spartobacteria*	0.10
				Xiphinematobacter	0.06
		Opitutae	0.05	*Opitutus*	0.05
(Planctomycetes, TM7, Chloroflexi, Cyanobacteria/ Chloroplast, Deinococcus)	1.20	*Phycisphaerae*	0.34	*Phycisphaera*	0.34
		TM7	0.30	*TM7_genera_incertae_sedis*	0.30
		Thermomicrobia	0.23	*Sphaerobacter*	0.23
		Chloroplast	0.09	*Chlorophyta*	0.09
		Deinococci	0.09	*Truepera*	0.09
		Others	0.15	Others	0.15
